# Anatomical Characteristics and Variation Mechanisms on the Thick-Walled and Dwarfed Culm of Shidu Bamboo (*Phyllostachys nidularia* f. *farcta*)

**DOI:** 10.3389/fpls.2022.876658

**Published:** 2022-05-24

**Authors:** Yujun Wang, Guirong Qiao, Jing Xu, Kangming Jin, Minyuan Fan, Yulong Ding, Qiang Wei, Renying Zhuo

**Affiliations:** ^1^State Key Laboratory of Tree Genetics and Breeding, Chinese Academy of Forestry, Beijing, China; ^2^Key Laboratory of Tree Breeding of Zhejiang Province, Research Institute of Subtropical Forestry, Chinese Academy of Forestry, Hangzhou, China; ^3^College of Plant Protection, China Agricultural University, Beijing, China; ^4^Bamboo Research Institute, Nanjing Forestry University, Nanjing, China

**Keywords:** wall thickness, internode length, fiber cell, parenchyma cell, transverse morphology, elongation growth, biomass

## Abstract

Stable culm variants are valuable and important material for the study of culm development in bamboo plants. However, to date, there are few reports on the mechanism of variation of these bamboo variants. *Phyllostachys nidularia* f. *farcta* (Shidu bamboo) is a bamboo variant with stable phenotypes such as a dwarf culm with a thickened wall. In this study, we systematically investigated the cytological characteristics and underlying mechanism of morphological variation in culms of this variant using anatomical, mathematical statistical, physiological, and genomic methods. The anatomical observation and statistical results showed that the lateral increase of ground tissue in the inner layer of culm wall and the enlargement of vascular bundles are the anatomical essence of the wall thickening of Shidu bamboo; the limited elongation of fiber cells and the decrease in the number of parenchyma cells longitudinally are probably the main causes of the shortening of its internodes. A number of genes involved in the gibberellin synthesis pathway and in the synthesis of cell wall components are differentially expressed between the variant and its prototype, *Ph. nidularia*, and may play an important role in determining the phenotype of internode shortening in Shidu bamboo. The decrease in gibberellin content and the content of the major chemical components of the cell wall of Shidu bamboo confirmed the results of the above transcriptome. In addition, the variation in culm morphology in Shidu bamboo had little effect on the volume of the culm wall of individual internodes, suggesting that the decrease in the total number of internodes and the decrease in dry matter content (lignin, cellulose, etc.) may be the main factor for the sharp decline in culm biomass of Shidu bamboo.

## Introduction

Woody bamboo is unique among plants because it has both the tissue morphology of herbaceous plants and the material properties of woody plants. In addition, the woody properties of bamboo culm and its rapid growth cycle give it great economic and ecological value (Gao et al., 2022). Therefore, the study of biological processes related to bamboo culm morphogenesis and underlying regulatory mechanisms have traditionally been a research focus of researchers in related fields.

The morphological development of the bamboo culm proceeds mainly in two typical stages. The first stage is the primary thickening growth of the shoot bud under the ground, which mainly driven by shoot apical meristem (SAM) (Wei et al., 2017, 2018; Wang et al., 2019); the second stage is the rapid growth of the young bamboo, which is driven by the intercalary meristem (Wei et al., 2019). Efforts have long been made to explore the regulatory mechanism, especially the molecular mechanism of bamboo culm in the above morphological development process (Peng et al., 2013a; Gamuyao et al., 2017; Li et al., 2018; Jin et al., 2021). However, a number of obstacles, such as the complex genomic background of bamboo plants and the long and uncertain sexual reproductive cycle (Peng et al., 2013b; Guo et al., 2019; Zheng et al., 2020, 2022), makes it difficult to deepen the relevant research. Fortunately, several bamboo variants have attracted people's attention in the recent years (Wei et al., 2017, 2018; Wang et al., 2018; Hu et al., 2020). Systematic analysis of the phenotypes of these bamboo variants, combined with comparative transcriptome analysis and other biological study methods, people can further investigate the genes responsible for regulating key traits, and also provides a new avenue to further explore the molecular basis and regulatory mechanism associated with bamboo morphogenesis. Currently, a number of researchers have made important breakthroughs in this way. For example, Wei et al. (2017) have found a set of genes that play an important regulatory role in the primary thickening growth process of bamboo by studying a thickened wall variant with abnormal development (*Phyllostachys edulis*, “Pachyloen”). Similar ideas were also used to find the main candidate genes regulating the fast growth of bamboo, and a bamboo variant with a slow-growing trait (*Pseudosasa japonica* var. *tsutsumiana*) played a key role (Wei et al., 2018). Therefore, the stable variants of bamboo can be used as an effective tool to better understand the process of culm morphogenesis. This research also makes us aware of the importance of discovering and further studying these valuable bamboo variants.

Shidu bamboo (*Ph. nidularia* f. *farcta*) is a stable variant with walls of varying thickness (Wang et al., 2019). It is an ideal material to study the formation of the transverse morphology of the culm wall. However, research on this bamboo species is currently very limited. In our previous study, we observed the tissue morphology of its shoot bud in the early developmental process (the primary thickening growth process), and identified the main factor affecting the morphological development and the underlying molecular basis in advance (Wang et al., 2019). However, the pattern of culm wall thickening in *Ph. nidularia* f. *farcta* is still unclear. In addition, a number of other morphological changes in the culm, such as the reduced culm diameter, shortened internode length, reduced total number of internodes, and significantly reduced fresh weight, suggest that there are other obstacles to the morphological development of the culm of *Ph. nidularia* f. *farcta*, that require further study to confirm and explain.

To address these questions, in this study, we systematically analyzed the tissue structure of the culm wall of *Ph. nidularia* f. *farcta*, as well as the cytological causes of the shortened internode and the underlying mechanism. We also investigated the main factors affecting the decline of biomass in the culm of *Ph. nidularia* f. *farcta* by using statistical analysis method and histochemical evidence.

## Materials and Methods

### Plant Materials

The bamboo culm samples of the variant (*Ph. nidularia* f. *farcta*) and primitive type (*Ph. nidularia*) involved in this study were collected in Libo city, Guizhou province, China (25°29′N, 107°51′E). The bamboo forests of the above two bamboo species were adjacent to each other, had which were primitive distribution and have the same growing conditions. The collection site was located in a humid monsoon climate in the middle subtropical zone. The average altitude of the bamboo forest area was 941 m and the mean annual air temperature was 18.3°C and the rainfall was 4,000 mm. To determine the standard bamboo, we first measured the diameter at breast height (DBH) of 30 bamboo culm of each species randomly, and calculated the average DBH. Then, 10 1-year-old bamboo culms of each bamboo species, with average DBH, were selected and cut from the base as standard samples to conduct the following experiments.

### Morphological Analysis and Determination of Biomass of Bamboo Culms

The length and diameter of the internodes, the thickness of the culm wall, and the total number of internodes of standard bamboo were measured. Then, the culm wall area in cross-section and the culm wall volume of each internode were calculated based on the above measured data. We also calculated the ratio of wall thickness and cavity diameter (wall-cavity ratio) of internodes from each part of the above two types of bamboo and the formula was as follows:


(1) $$\begin{array}{r} R\;=\;\frac{WT\;\times 2}{D\;-\;WT\;\times \;2} \end{array}$$

Where R was the wall cavity ratio, WT was the wall thickness of bamboo culm (mm), and D was the internode diameter (mm).

To measure the biomass of bamboo culms, we first measured the total fresh weight of each standard bamboo after removing the branches and leaves and then separated an internode from the top, middle, and bottom of each standard bamboo culm. After marking the internodes, 400 g of fresh culm samples were collected from each internode were and brought back to the laboratory. These samples were dried in an oven at 130°C to a constant weight, then the dry weight of each sample was weighed and the moisture content and dry weight of each standard culm were calculated. SPSS statistics version 17.0 was used for difference analysis of the data.

### Microscopic Observation of the Culm Wall

To observe the tissue morphology of the culm wall in cross section. The samples were first separated from the different positions of the standard bamboo culm, and then further cut into smaller bamboo strips with a size of about 3 × 1 × 1 cm. At least 3 samples were collected from each culm position of the two bamboo species, representing at least 3 biological replicates. These samples were fixed with the FAA solution (70% ethanol, formalin, and acetic acid, 18:1:1, v/v/v) for 3 days and then softened in 70% alcohol-glycerin (1:1, v/v) for 2 weeks. After embedding in PEG2000, the samples were cut into 20-micrometre cross sections using a Microtome 860 (American Optical Corporation, New York, USA). These sections were stained with Safranin O and aqueous Alcian Blue and then observed under a Leica DM2500 light microscope (Leica, Wetzlar, Germany) (Wang et al., 2016).

Based on these cross-sectional photographs of the bamboo culm, we measured the density of the vascular bundles, the size, and the area ratio of the individual wall tissue components of the two bamboo species using ImageJ version 1.48 software.

For the analysis of the longitudinal tissue morphology of the culm wall, samples from different culm positions of standard bamboo were divided into pieces of approximately 1.5 cm^3^. After a series of pre-treatments, including fixation of the samples (with FAA solution), and softening (70% alcohol and glycerin), which was similar to that of the pervious section. The longitudinal section of the samples was smoothed with a sharp blade. After dehydration and drying, these samples were sprayed with gold and observed under the JEOL JSM-6300 scanning electron microscope (JEOL, Tokyo, Japan). A detailed protocol can be found in a study by Wang et al. (2019).

### Analysis of Cell Morphology

To observe the morphological characteristics of the fiber cells, the samples from different culm positions were cut into small sticks of about 2 cm in length. Then, these samples were immersed in Jeffrey solution (10% aqueous nitric acid/10% aqueous chromic acid, 1:1), and heated at 60°C for 9 hours. After being washed with distilled water, the samples were placed in 75% alcohol and examined by tablet compression. At least 150 fiber cells of each sample were observed with the light microscope (Leica DM2500) and the cell morphological indices, including fiber length, width, lumen diameter, and cell wall thickness, were immediately measured using Leica QWin version 3 software.

From the scanning electron micrographs of the longitudinal section of the culm wall, we observed the morphology of the parenchyma cells and measured the length and width of the parenchyma cells using ImageJ version 1.48 software.

Statistics and difference analysis of the data were performed using the Microsoft Office Excel 2013.

### Re-analysis of the Transcriptome Data

Based on the transcriptome data of shoot buds of *Ph. nidularia* f. *farcta* and its primitive type *Ph. nidularia*, whose data were measured and published in 2019 (Wang et al., 2019) (Accession Number SRR7796323–SRR7796330). We reanalyzed and screened the differentially expressed genes (DEGs) related to gibberellin synthesis pathway and cell wall components using MapMan visual analysis software (version 3.5.1 *R*^2^). A detailed method for data processing was described in a study by Wang et al. (2019).

### Ribonucleic Acid Extraction and Quantitative Real-Time PCR

Five bamboo shoots of *Ph. nidularia* f. *farcta* and its primitive type in the fast growth phase with a height of 15 cm were collected and the shoot sheath was removed. Total RNA extraction, purification, and the complementary DNA (cDNA) synthesis, as well as the quantitative PCR (qPCR) were performed as described in a study by Jin et al. (2020). The relative abundance of the target gene was calculated from the 2^−Δ*Cq*^ value between itself and the reference gene [tonoplast intrinsic protein 41 (TIP41)] (Fan et al., 2013). The specific primers used for qPCR were designed by Primer Premier 5 and listed in Supplementary Table S1.

### Quantification of Gibberellins

For the detection of the gibberellins (GAs), ~9 bamboo shoots of each species were collected at 15 cm height during the fast growth phase. Mix 3 shoots at a time to form a sample. A total of three biological replicates of the two bamboo species were used to determine the content of four GAs (GA1, GA3, GA4, and GA7). These samples were ground into powder in liquid nitrogen, and 200 mg of powder for subsequent determination. The endogenous hormones were extracted in isopropanol-water-hydrochloric acid solution. Subsequently, the hormone content in the sample was determined by high-performance liquid chromatography (Agilent 1290) and tandem mass spectrometry (AB Sciex QTRAP 6500), with internal standard substances added during extraction to correct the detection results.

### Determination of Cell Wall Composition

The culm samples for chemical composition determination were divided and mixed from the top, middle, and bottom parts of the standard bamboo culm. A total of 20 g culm samples from each position were uniformly mixed, dried, and grounded into powder to determine the basic chemical constituents. The cellulose content was measured using a referenced method (Wang, 2006). The sample was first weighed to the nearest 100 mg, and then heated and hydrolyzed under acidic conditions. The cellulose content was finally determined and calculated by anthrone-sulfuric acid colorimetry. The content of hemicellulose was determined by 3,5-Dinitrosalicylic acid (DNS) reducing sugar method. The detailed detection steps were described by Chen (2002). For the detection of lignin content, following the method described in a study by Pochinok et al. (1981), 72% concentrated sulfuric acid was first used to remove cellulose and then the lignin content was calculated indirectly by an oxidation reaction.

## Results

### Morphological Characteristics of the Culm of Shidu Bamboo

**Figure 1:**
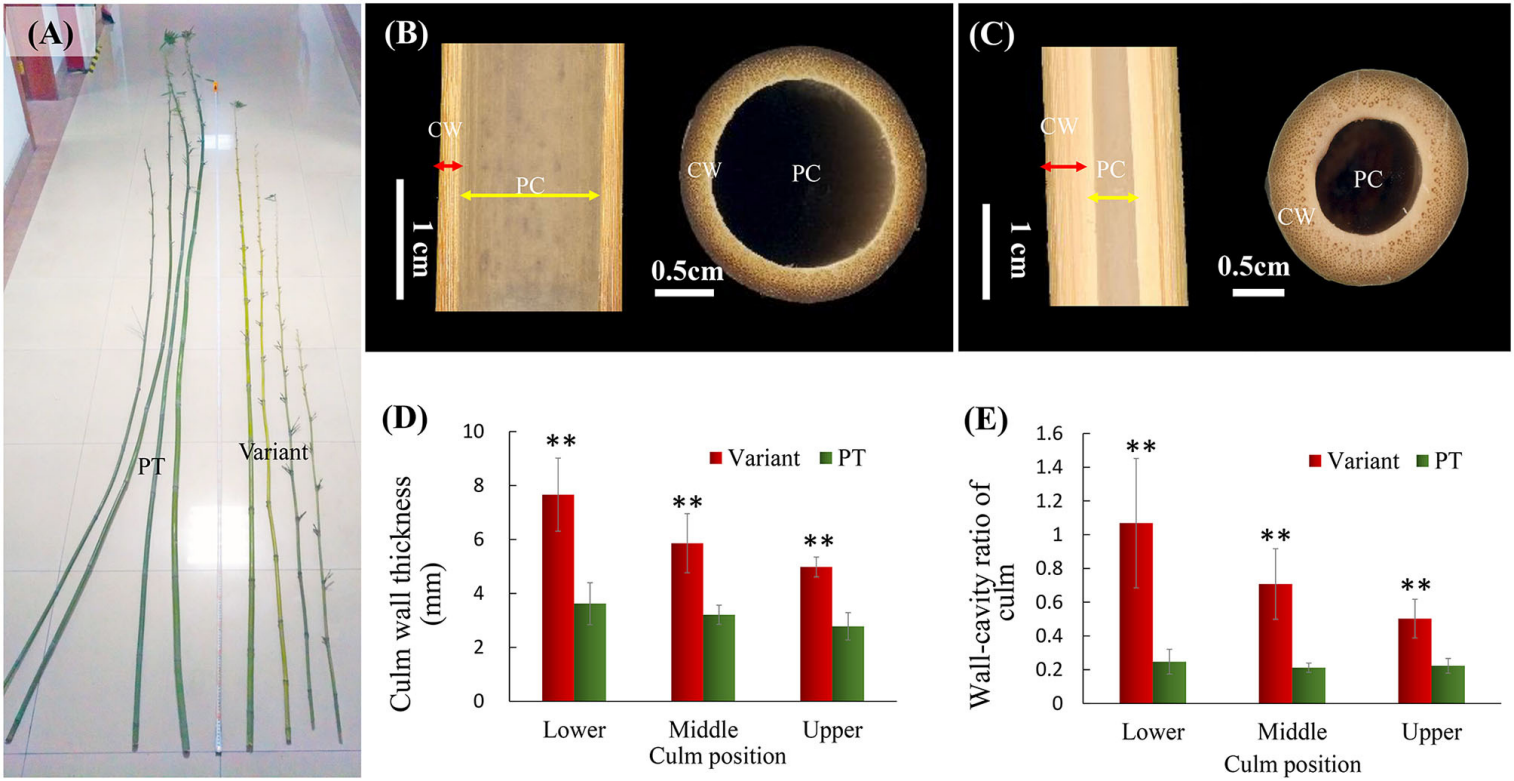
Morphological characteristics of the culm of Shidu bamboo (the variant plant) and the prototype plant (PT). **(A)** Appearance of the culm of the variant and the PT. **(B)** Iternal morphology of the culm in the PT plant and **(C)** the variant. CW, the culm wall; PC, the pith cavity. **(D)** Culm wall thickness of the variant and the PT plant at different culm positions. **(E)** The ratio of wall thickness and cavity diameter of the variant and the PT plant at different culm positions. ^**^P < 0.01.

Compared to the prototype plant, the culm of Shidu bamboo (*Ph. nidularia* f. *farcta*) was much shorter and smaller but straighter (Figure 1A). The internal morphology of the culm showed that its straight appearance was related to the thickened culm wall (Figures 1B–E). Based on the cross-section and longitudinal-section of the culm wall, we obviously found that the culm wall in Shidu bamboo (Figure 1B) was significantly thicker than that in the prototype plant (Figure 1A), while its diameter of pith cavity was narrower than that in the primary type (Figures 1A,B). Further quantitative results showed that the wall thickness of each part of the variant was significantly greater than that of the prototype plant, especially in the lower part of the culm, and whose thickness reached twice that of the prototype plant (Figure 1C). Furthermore, the ratio of the wall thickness to the diameter of the pith cavity of the variant was two to five times greater than that of the prototype plant (Figure 1D).

### Anatomical Characteristics of the Culm Wall in Shidu Bamboo and Its Prototype Plant

**Figure 2:**
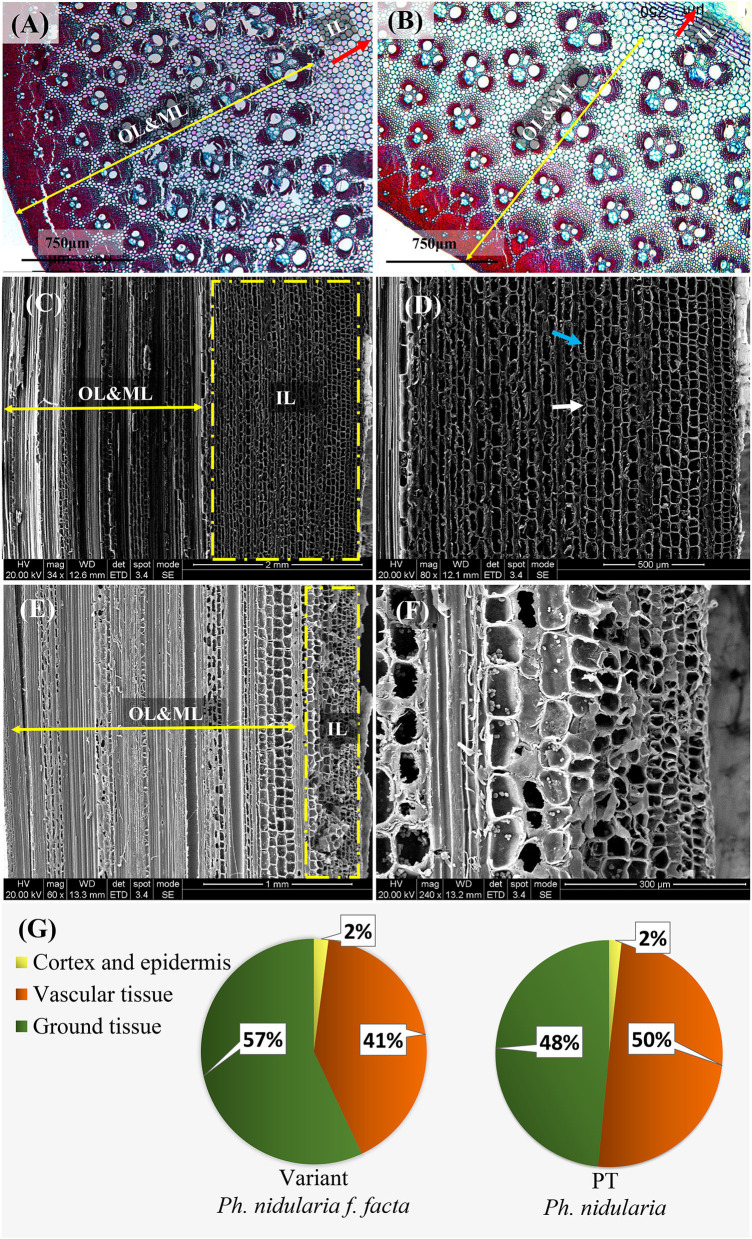
Anatomical characteristics of the culm wall and the ratio of wall components in cross-section in Shidu (the variant) and its prototype plant (PT). The anatomical morphology in cross-section of the culm wall of the variant plant **(A)** and its PT plant **(B)**. OL and ML, the outer layer and the middle layer of the culm wall, respectively corresponding to the direction and boundary indicated by the yellow double arrow; IL, the inner layer of the culm wall, corresponding to the direction and area indicated by the red arrow. **(C)** Anatomical morphology in vertical section of the culm wall of the variant plant. The yellow dotted box shows the thickening of the tissue in the inner layer of the culm, further magnified in **(D)**. The long cell indicated by blue arrows and the short cell indicated by white arrows indicate that these thickening tissue belongs to the ground tissue in the wall component. **(E)** Anatomical morphology in vertical section of plant culm wall of the PT. The yellow dotted box shows the normal anatomical morphology in the inner layer of the bamboo culm and the enlarged view was shown in **(F)**. **(G)** The proportion of the tissue of the wall components in cross-section of the culm in these two bamboo species.

The outer and middle layers of the culm wall both the Shidu bamboo (Figure 2A) and its prototype plant (Figure 2B), had a similar anatomical structure, which was a typical transverse anatomical feature of the bamboo culm, i.e., the outer layer of the bamboo culm consisted of epidermis and cortex, while the middle side of the culm had, vascular tissue scattered in the ground tissue. In the inner layer of the culm wall, the wall tissues of the prototype plant consisted entirely of multiple layers of parenchyma cells (~12 layers of parenchyma cells could be counted in longitudinal section) (Figures 2E,F). An area named “transition layer” could be observed, consisting of several rows of cells with mostly radially and axially shortened cells. This was also a typical morphology of the culm wall structure in common bamboo plants. However, the typical characteristics of the transition layer were not evident on the culm of Shidu bamboo (Figure 2D). Furthermore, greatly enlarged parenchyma cell layers were observed in this area, resulting in this area being more than twice as thick as its prototype plant (Figures 2C,E). Besides, the long and short cells belonging to the ground tissue of bamboo plants were found in these enlarged parenchyma cells (Figure 2D). The area proportion of the epidermis, cortex, and vascular bundle, and ground tissue in the cross-section of the culm wall was further analyzed (Figure 2G). Consistent with the anatomical observation, the area of ground tissue in the Shidu culm reached 57%, which was much larger than that of the prototype (48%). In contrast, the proportion of vascular tissue in the Shidu culm (41%) was relatively smaller than that of the prototype plant (50%), whereas the proportion of epidermis and cortex was similar in both.

### Morphological Comparison of Vascular Tissue Between Shidu Bamboo and Its Prototype Plant

**Figure 3:**
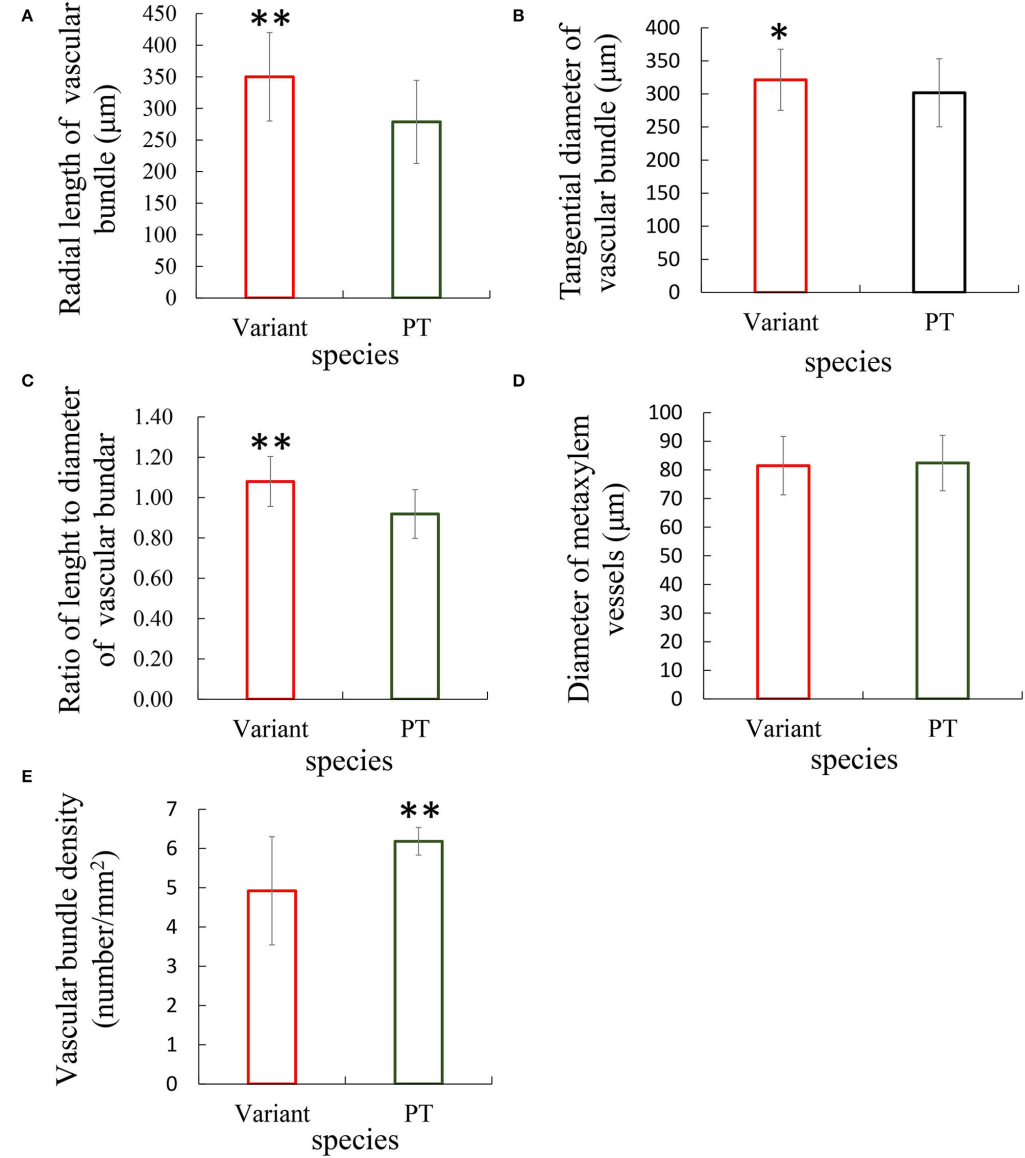
Comparative analysis of the morphology and density of vascular bundles between Shidu bamboo (variant) and its prototype plant (PT). **(A–D)** Morphological parameters of the vascular bundles of the variant and the PT plant, including **(A)** radial length, **(B)** tangential diameter, **(C)** the ratio of length and diameter, and **(D)** the diameter of metaxylem vessels in the vascular bundle. **(E)** The density of vascular bundle in culm wall of the variant and its PT plant. *P < 0.05, **P < 0.01.

The anatomical analysis revealed that the transversely enlarged parenchyma cell layer in the inner layer of the culm mainly contributed to the thickening of the culm wall of Shidu bamboo. On the other hand, the relative proportion of vascular tissue, which may result in masking the contribution of vascular tissue. To verify whether the vascular tissue in Shidu bamboo contributes to the thickening of the culm wall, the size and density of vascular bundles in the cross-section of the culm wall of Shidu bamboo and the prototype plant were measured and compared (Figures 3A–E). The results showed that the size of the vascular bundle of Shidu bamboo was significantly larger than that of the prototype plant in terms of both radial length and tangential diameter, and the ratio between length and diameter (Figures 3A–C), while no significant difference was found in the diameter of the metaxylem vessels in the vascular bundle between the two plants (Figure 3D). However, the density of the vascular bundle of Shidu bamboo was significantly lower than that of the prototype plant (Figure 3E).

### Morphological Analysis of Cells in the Culm Wall

In addition to the thickening of the culm wall, we also observed that the culm of Shidu bamboo was dwarfed (Figure 1A), so we hypothesized that its internodes were restricted in their elongation growth. To verify this conjecture, the morphology and the size of fibrous cells and parenchyma cells in the culm wall of Shidu bamboo and the prototype plant were analyzed and compared (Tables 1, 2). The comparative analysis showed that the fiber cells in the culm of Shidu bamboo were significantly shorter in length but wider in width than those of the prototype plant, resulting in a significant decrease in the length-to-width ratio of fiber cells in Shidu bamboo (Table 1). Furthermore, the lumen diameter of the fiber cell in Shidu bamboo was significantly larger than in the prototype plant, but the thickness of the cell wall was smaller (the difference did not reach a significant level), resulting in a significant larger ratio of cell wall thickness to lumen diameter of the fiber cell in Shidu bamboo (Table 1). In contrast to the difference in fiber cell characteristics, the parenchyma cell in the inner layer of Shidu culm was significantly longer but narrower in width when compared with the same position of the prototype culm, resulting in a significantly higher length–to-width ratio of the parenchyma cell in Shidu bamboo than that of its prototype plant (Table 2).

**Table 1:** Fiber characteristics of the wall thickness variant and its prototype plant.

**Species**	**Index**	**Length/μm**	**Width/μm**	**Length-width ratio**	**Wall thickness/μm**	**Lumen diameter/μm**	**Wall-Lumen ratio**
The variant	Max	3,293.20	30.66	202.92	13.20	21.04	18.88
	Min	477.72	11.02	29.31	2.67	1.34	0.28
	Mean	1,696.05 ± 405.67a	19.26 ± 4.18b	91.55 ± 27.61a	6.25 ± 2.32a	6.76 ± 3.77b	2.90 ± 2.77a
The prototype	Max	3,152.74	25.96	211.42	11.39	11.76	10.49
	Min	1,010.72	9.45	50.53	3.07	1.38	0.58
	Mean	1,901.80 ± 402.73b	17.46 ± 3.34a	112.62 ± 30.99b	6.91 ± 1.60a	3.65 ± 1.49a	4.31 ± 1.77b

*Different letters in the same column mean significant at 0.05 level*.

**Table 2:** Size of the parenchyma cell of the wall thickness variant and prototype plant.

**Species**	**Index**	**Length/μm**	**Width/μm**	**Length-width ratio**
The variant	Max	207.71	61.71	6.73
	Min	14.61	26.08	0.43
	Mean	92.80 ± 37.27b	43.58 ± 6.88a	2.18 ± 1.00b
The prototype	Max	149.36	64.96	5.11
	Min	17.86	24.41	0.35
	Mean	71.18 ± 22.99a	45.40 ± 11.01b	1.67 ± 0.75a

*Different letters in the same column mean significant at 0.05 level*.

### Alterations of Gibberellin and Cell Wall Components and the Underlying Molecular Evidence

**Figure 4:**
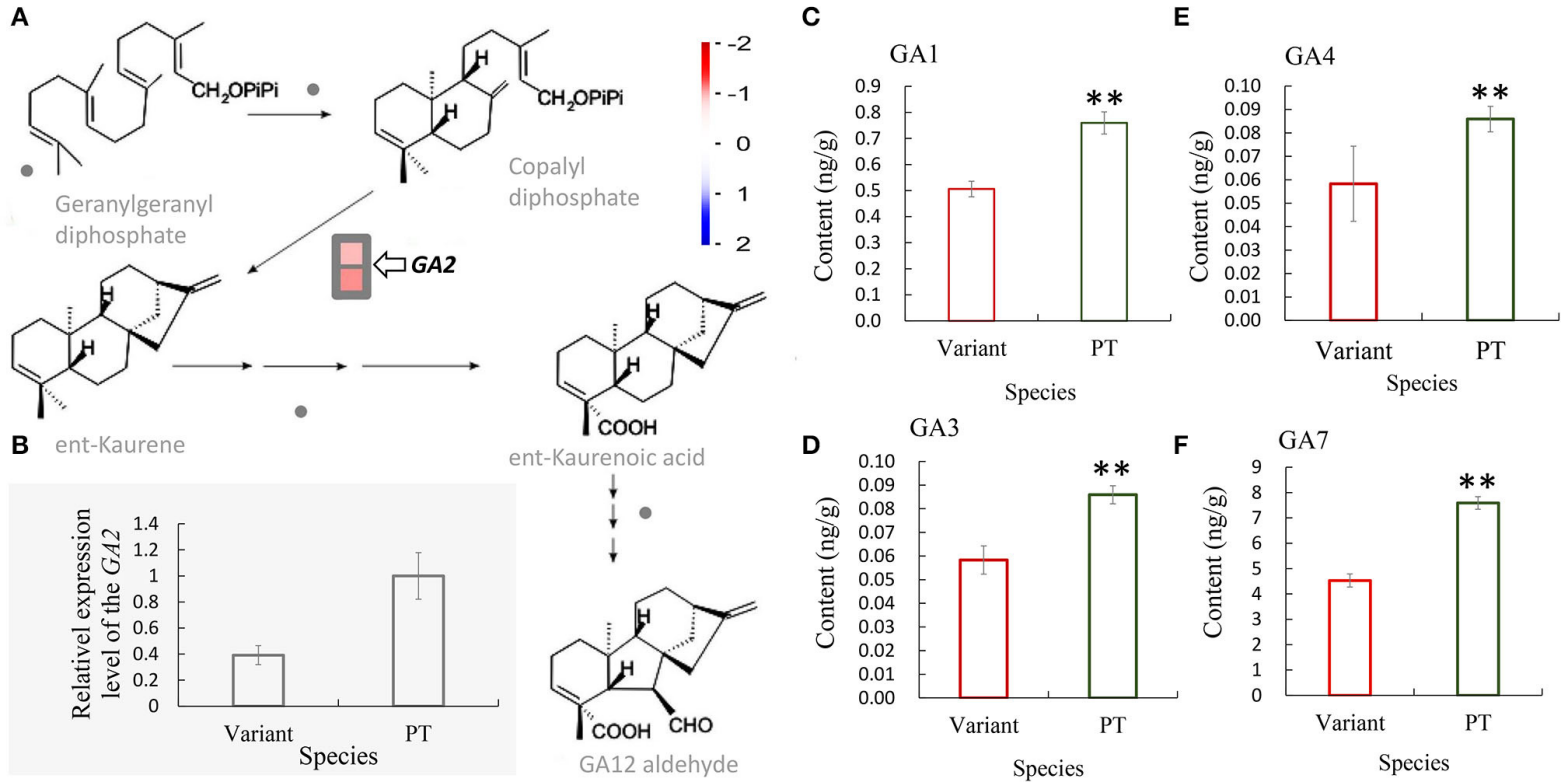
Decreased expression of the *GA2* gene in Shidu bamboo (the variant) might result in a decrease in the content of at least four gibberellins. **(A)** Transcriptome analysis showed that the *GA2* gene, which encodes an enzyme that plays a key role in the production of an early intermediate (*ent*-kaurene) in gibberellin (GA) biosynthetic pathways, was significantly downregulated in the variant. **(B)** Quantitative real-time PCR (qRT-PCR) analysis of the *GA2* gene of the variant plant at the fast growth stage. Data are means ± SD. **(C–F)** Content of four gibberellins: **(C)** GA1, **(D)** GA3, **(E)** GA4, and **(F)** GA7 of the variant plant (variant) and the prototype plant (PT) at the fast growth stage. **P < 0.01.

Gibberellin plays an important role in promoting internode elongation of gramineae. In our previous study, we sequenced and analyzed the transcriptome of the shoot bud of Shidu bamboo and its prototype plant during the primary thickening growth and found 844 differentially expressed genes (DEGs). Among them, two genes related to gibberellin synthesis pathway were downregulated in Shidu bamboo (Wang et al., 2019). By further visualizing the relative data, we found that these two DEGs all encode a protein with *ent*-kaurene synthase B activity and play an important role in the synthesis of *ent*-kaurene, which is an early intermediate in the synthesis pathway of gibberellin (Figure 4A; Supplementary Table S2). We performed a qRT-PCR assay to check the expression level of this gene in the shoot during the fast growth phase. The result was consistent with the result of our previous transcriptome analysis (Figure 4B). Subsequently, we also determined the levels of four gibberellins, namely, GA1, GA2, GA4, and GA7 in the young shoot at the fast growth stage. The results showed that the contents of the above four gibberellins were significantly lower in the young shoots of Shidu than in the young shoots of the prototype (Figures 4C–F).

**Figure 5:**
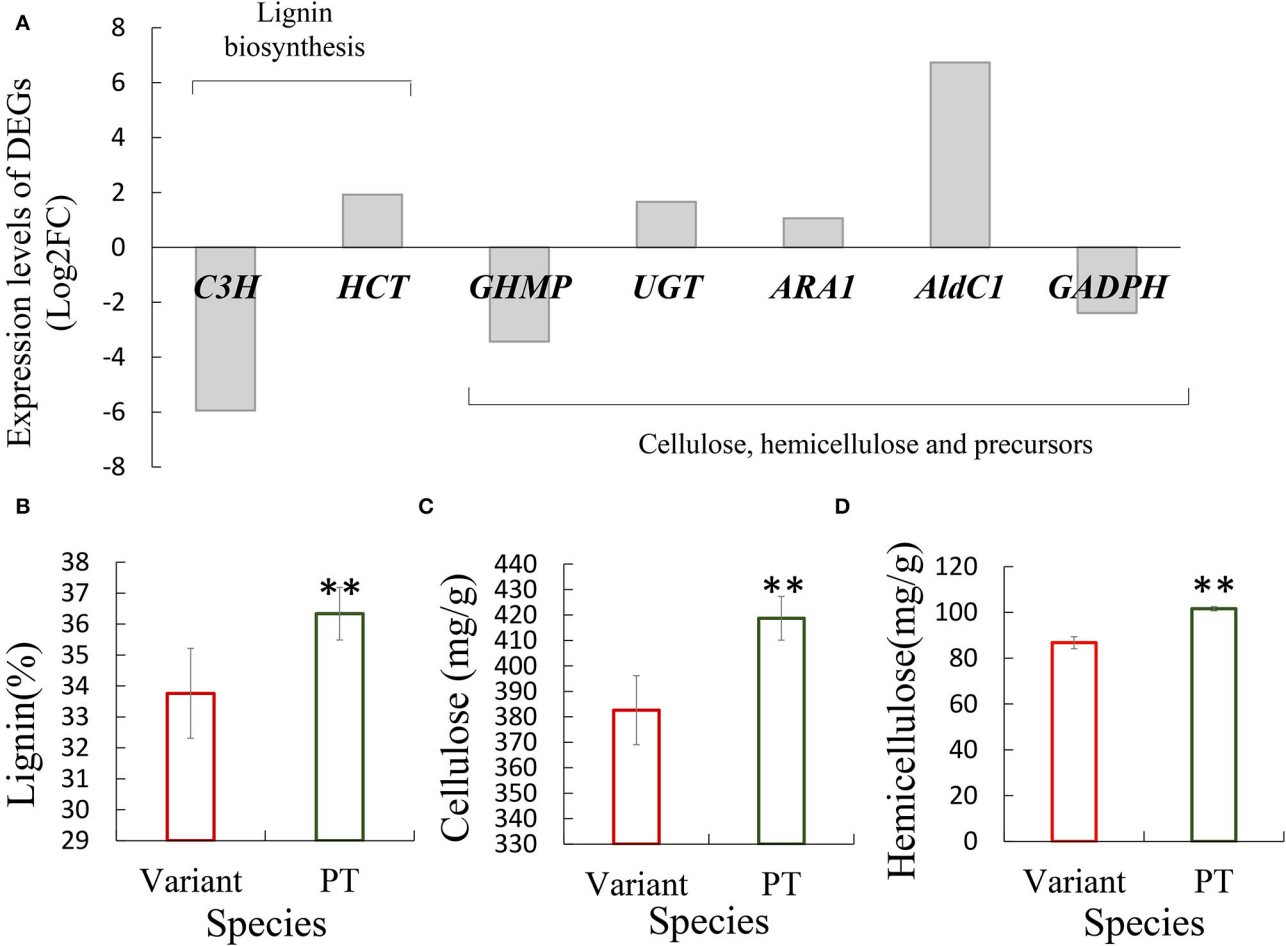
Differentially expressed genes (DEGs) related to the synthesis of cell wall components affected the content of major chemical components in the culm wall of Shidu bamboo. **(A)** Expression level of DEGs. **(B–D)** Comparison of the content of the content of major chemical components, including lignin **(B)**, cellulose **(C)**, and hemicellulose **(D)** in the culm wall between Shidu bamboo (variant) and its prototype plant (PT). **P < 0.01.

In addition, based on the measurement results of cell wall thickness of fiber cells in Shidu culm, we found that a number of genes related to the biosynthesis of cell wall components, including *C3H* and *HCT* involved in lignin biosynthesis and other involved in the synthesis and regulation of cellulose, hemicellulose, or related precursors, were differentially expressed in Shidu bamboo. These DEGs could also affect normal cellular activities in the internode elongation process (Figure 5A; Supplementary Figure S1; Supplementary Table S3). To verify this result, we determined and compared the contents of lignin, cellulose, and hemicellulose in the culm of Shidu bamboo and its prototype plants. The quantitative results showed that the content of the above three chemical components was significantly lower in the culm wall of Shidu than in the prototype plant (Figures 5B–D).

### Comparative Analysis of Internode Morphology, Culm Wall Volume, and Culm Biomass Between Shidu Bamboo and the Prototype

To investigate the effects of these morphological variations on the internode wall volume and culm biomass of Shidu bamboo, we first analyzed the morphological indices of the internode. As shown in Table 3, the internode of Shidu bamboo were significantly shorter and thinner compared to the prototype plant. Nevertheless, the thickened culm wall (up to twice the wall thickness of the prototype) considerably increases the culm wall area in the cross-section of Shidu bamboo, which compensates for in loss of wall volume caused by the reduction in internode length and diameter and finally results in the wall volume of the internode of Shidu being even larger than that of the prototype, although the difference was not significant. However, the total number of internode and the biomass of the culm of Shidu bamboo (the dry weight) were significantly lower than those of the prototype, especially the culm biomass (the dry weight of the culm of Shidu bamboo was less than half that of the prototype).

**Table 3:** Overall morphological index of internodes and culm biomass of Shidu bamboo and its prototype plant.

**Species**	**Index**	**IL (cm)**	**ID (cm)**	**WT (mm)**	**CWA (cm^**2**^)**	**WVI (cm^**3**^)**	**TIN**	**DW (g)**
The variant	Mean	25.77b	2.97b	6.42a	4.68a	120.66a	30.60b	878.11b
	SD (±)	5.73	0.23	0.62	0.55	31.09	8.04	222.28
The prototype	Mean	33.56a	3.58a	3.34b	3.40b	115.41a	38.57a	2,281.61a
	SD (±)	4.18	0.19	0.44	0.48	30.93	4.86	444.19

*Different letters in the same column mean significant at 0.05 level*.

*IL, internode length; IW, internode diameter; WT, Internode wall thickness; CWA, culm wall area of internode cross section; WVI, internode wall volume; TNI, total internode number; DW, dry weight of culm*.

## Discussion

Variations with thick-walled features have attracted more attention in bamboo plants because they contribute to the utilization of bamboo material. However, in this type of bamboo variation, the thickening of the culm wall was often accompanied by a reduction in the overall size of the culm, such as in *Ph. edulis* “Pachyloen” and *Ph. heteroclada* f. *solida* (Wei et al., 2017; Yue et al., 2017; Hu et al., 2020). In addition, comprehensive study on the anatomical characteristics and variation mechanism of culm variation of these valuable bamboo variants was still limited. As a member of thick-walled bamboo variants in thick wall type, *Ph. nidularia* f. *farcta* (Shidu bamboo) has higher scientific study value due to its stable variant phenotype and distinct origin (Figures 1A,B). We studied the morphology of its shoot apical meristem, the developmental process of its shoot bud, and its transcriptome in our previous study (Wang et al., 2019). In this study, we systematically analyzed the anatomical essence of Shidu bamboo wall thickening, and focused on the cytological reasons and potential mechanisms underlying the limited internode elongation. We also examined the effects of these morphological variations on internode wall volume and culm biomass of Shidu bamboo.

### The Thickening of Culm Wall of *Ph. nidularia* f. *farcta* Was Mainly Due to the Increase of Ground Tissue in the Inner Layer of the Culm

The tissue component of the culm all usually showed a certain distribution pattern in the cross-section of the bamboo culm. The outermost of culm consisted of epidermis and cortex; the middle layer consisted of ground tissue and the vascular tissue (Liese, 1985, 1998); the innermost part consisted of parenchyma cells, only including a few layers of ground tissue and a transition layer that extended into the pith cavity (Liese and Schmitt, 2006). The middle layer was the main part of the bamboo culm, determining the thickness of the culm wall. From the cross-section of the culm wall, it could be seen that the culm wall components of Shidu bamboo and its prototype were consistent with the distribution pattern described above (Figures 2A,B). Interestingly, although the size of the vascular bundles was significantly larger in Shidu compared to its prototype, the thickness of the middle layer of the culm did not obviously increase (Figures 2C, 3A–C). The decrease in the density of the vascular bundles also indicates that the vascular tissue in the culm walls of Shidu bamboo did not increase (Figure 3E). On the contrary, a large number of proliferative parenchyma tissues were observed in the inner wall layer of Shidu bamboo, making the thickness of this part more than several times that of the corresponding part of the prototype plant (Figures 2C,E). Furthermore, two types of parenchyma cells (the long parenchyma cells and the short parenchyma cells) were found in the tissues, indicating that the proliferative parenchyma tissue belongs to the ground tissue of the bamboo culm (He et al., 2002). Therefore, from the above results, it can be inferred that the increase in the wall thickness of the variant was mainly caused by the proliferation of the ground tissue in the inner layer of the culm wall. The statistical results of the area percentage of the ground tissue (57%) also confirmed this ratio (Figure 2G). According to the statistics of Liese (1985), the ground tissue and vascular tissue each account for about 50% of the bamboo culm, which is consistent with the statistical results of the prototype plant culm in this study.

### Limited Elongation of Fiber Cells and the Decrease in the Number of Parenchyma Cells Longitudinally Result in the Shortening of the Internodes of Shidu Bamboo

The elongation of culm internode was caused by the synergistic growth of vascular tissue and parenchyma in bamboo plants. At the cellular level, we found that the length and cell wall thickness of fiber cells of Shidu bamboo were all shorter and thinner than those of the prototype plant (Table 1), indicating that the elongation process of these fiber cells was inhibited. Similar morphological characteristics of the fiber cells were also observed in another dwarf bamboo variant (Wei et al., 2018). Interestingly, the parenchyma cells in the culm of Shidu bamboo became significantly longer and narrower than those of the prototype plant, which were completely opposite variation characteristics to the fiber cells in the culm of Shidu bamboo (Table 2). Combined with the features of shortening internode length in Shidu bamboo, it could be concluded that the number of parenchyma cells in the internodes of the variant was actually reduced in the axial direction compared with the internodes of its prototype plant. The longitudinal proliferation of parenchyma cells in the internode resulted from cell division of the intercalary meristem, which is located at the base of internode during elongation growth of the internode (Wei et al., 2019). It could also be concluded that the division ability of the meristem of Shidu bamboo was inhibited during the process of internode elongation. The above results also explain the phenomenon of internode shortening in Shidu bamboo at the cellular level.

### The Decrease in Gibberellins Content and Major Chemical Constituents of the Cell Wall of Shidu Bamboo May Have Affected the Elongation of Fiber Cells and the Activity of the Intercalary Meristem

Numerous of studies have shown the that metabolic pathways of endogenous plant hormones, including gibberellin, auxin, brassinosteroid, strigolactone, etc., as well as the metabolic pathways related to cell wall development, are closely associated with internode growth (Thomas and Sun, 2004; Lao et al., 2013; Tong et al., 2014; Han et al., 2016; Nagai et al., 2020; Zeng et al., 2022). Transcriptome results from our previous studies showed that genes involved in the gibberellin synthesis pathway that were differentially expressed in Shidu bamboo shoot buds. Further analysis showed that the *GA2* (GA requiring 2) that plays an important role in the early stage of gibberellin synthesis (Figure 4A), was significantly down-regulated in the Shidu bamboo shoot at the fast growth stage (Figure 4B), suggesting that the changes in the expression pattern of the *GA2* gene might affect the elongation growth of internodes of Shidu bamboo. Furthermore, the levels of four gibberellins (GA1, GA3, GA4, and GA7) were all significantly lower in Shidu bamboo than in the prototype plant (Figures 4C–F), further confirming the molecular cause of the limited elongation growth in Shidu internodes. The vascular tissue (fiber cells, xylem cells, and phloem cells) has already differentiated from the shoot apical meristem in the primary growth stage of bamboo shoot. Therefore, during the process of internode elongation, the cells of the vascular tissue mainly are proceeding elongation rather than division, whereas the parenchyma cells, due to their shorter length, must divide in sufficient numbers from the intercalary meristem to cooperate with the elongation of the vascular tissue (Wei et al., 2019). Therefore, we speculated that the reduced content of gibberellins should initially affect the elongation growth of the vascular tissue and the division ability of intermediate meristem in the internode elongation process of Shidu bamboo. The regulatory effect of gibberellin on the activity of the stem intercalary meristem of gramineae was also confirmed (Nagai et al., 2020). The cytological observation results of this study also support the above speculation. Therefore, we further speculated that the significant increase in the length of parenchyma cells in *Ph. nidularia* f. *farcta* was more of a passive result, i.e., the insufficient number of parenchyma cells can only be elongated with the rapidly elongated vascular tissue.

The changes in cell wall structure and components were another important factor affecting cell elongation (Scheible and Pauly, 2004; Coomey et al., 2021; Li et al., 2021; Wang et al., 2021). In Shidu bamboo, corresponding signs, such as the thinning of its fiber cell wall and the decrease in lignin, cellulose, and hemicellulose content in the culm wall compared with the prototype plant (Table 1; Figures 5B–D), indicate that its cell wall exhibits abnormal development. Some genes reported to play important roles in cellulose synthesis, such as the GHMP kinase family (*GHMP*) gene (Bar-Peled and O'Neill, 2011), or genes regulating lignin synthesis, such as *Coumarate*-3-hydroxylase (*C3H*) (Xu et al., 2009), as well as other genes related to the regulation of cell wall precursors, were significantly down-regulated in the variant (Figure 5A; Supplementary Table S3); this could lead to an abnormal development of cell wall in Shidu bamboo. For example, the *C3H* encodes a key enzyme, that plays an important role in the early stage of lignin synthesis, especially in the synthesis pathway of S and G monolignols (responsible for catalyzing p-coumaroyl CoA to caffeoyl-CoA) (Barros et al., 2019). Down regulation of its expression level leads to a maximal reduction of lignin content in *Medicago* (Chen and Dixon, 2007). In addition, another gene associated with lignin synthesis, shikimate hydroxycinnamoyl transferase (*HCT*) gene, which has been reported to play an important role in a parallel metabolic pathway with the *C3H* gene, was significantly upregulating in the variant. This result suggests that there may be a compensatory effect between the two parallel metabolic pathways mentioned above. It also reported that the expression of *C3H* gene was negatively correlated with the related genes in its parallel pathway in *Panicum virgatum* cells (Rao et al., 2017).

### Internode Morphological Variation of Shidu Bamboo Increased Its Internode Wall Volume, but Did Not Promote Its Culm Biomass

The increase in wall thickness and the decrease in culm size (the dwarf of the culm and the decrease in culm diameter) have opposite effects on culm wall volume and biomass. In the case of Shidu bamboo, the above variations have complementary effects on the culm wall volume of its individual internodes, so that its internode wall volume shows no significant difference compared to the prototype and even exceeds the prototype in mean value (Table 3). However, culm biomass showed a large difference between the variant and prototype plants (the culm dry weight of the variant was less than half that of the prototype plant). The reduction in the total number of internodes is likely to be one of the important factors leading to this result. Another important factor for the decrease in culm biomass might be related to the significant decrease in cellulose and lignin content in the culm wall of Shidu bamboo (Figures 5B–D). This is because the content of these chemical components accounts for over 90% of the total mass of bamboo culm (Li et al., 2007). Furthermore, some studies have shown that downregulation of *C3H* or *HCT* can significantly reduce plant biomass, with a maximum decrease of 40% (Reddy et al., 2005; Chen and Dixon, 2007), providing a molecular clue to the reason for the decrease in culm biomass of Shidu bamboo.

In summary, culm wall thickening in Shidu bamboo occurs mainly in the inner layer of the culm, resulting in transverse proliferation of the ground tissue, and is associated with a change in the proportion of each culm wall component in the cross-section of culm wall. In conjunction with our previous study results, it shows that this morphological change occurred mainly in the primary thickening growth process, which was mainly due to the abnormal cell division and differentiation of the shoot apical meristem; at the fast growth stage, the lower gibberellin level and abnormal cell wall development maight affect the elongation activity of cells in the internodes of Shidu bamboo, while the reduction in the division ability of the intercalary meristem further restricted the elongation of the internode and eventually led to the shortening of its internode length. A number of genes involved in the gibberellin synthesis pathway and the cell wall component synthesis pathway, such as *GA2, C3H*, and *HCT*, were differently expressed between Shidu bamboo and the prototype plant, which possibly leading to the limited elongation of the internode of Shidu bamboo. Variation in internode morphology had little effect on the average culm wall volume of Shidu bamboo internodes. The reduction in the total number of internodes and the content of the major chemical components of the culm wall could be the main causes for the significant decrease in culm biomass of Shidu bamboo.

## Data Availability Statement

The datasets presented in this study can be found in online repositories. The names of the repository/repositories and accession number(s) can be found in the article/Supplementary Material.

## Author Contributions

QW and RZ conceived this study. YW and GQ designed the experiments, interpreted the results, and wrote the manuscript. JX, KJ, and MF performed the experiments and analyzed the data. YD provided technical guidance for the experiment. All authors have read and approved the final submission of the manuscript.

## Funding

This study was funded by the China Postdoctoral Science Foundation (Grant No. 2020M670528) and the National Key Research and Development Programme of China (Grant No. 2021YFD2200504).

## Conflict of Interest

The authors declare that the research was conducted in the absence of any commercial or financial relationships that could be construed as a potential conflict of interest.

## Publisher's Note

All claims expressed in this article are solely those of the authors and do not necessarily represent those of their affiliated organizations, or those of the publisher, the editors and the reviewers. Any product that may be evaluated in this article, or claim that may be made by its manufacturer, is not guaranteed or endorsed by the publisher.
