# Supplementary material for: Anatomical Characteristics and Variation Mechanisms on the Thick-Walled and Dwarfed Culm of Shidu Bamboo (Phyllostachys nidularia f. farcta)
Source: Front Plant Sci. 2022 May 24;13:876658. doi: 10.3389/fpls.2022.876658 (PMC9171372; doi:10.3389/fpls.2022.876658)
Supplement: Supplementary file 2 [file Data_Sheet_1.docx]

Supplementary Material

# Supplementary Figures and Tables

**Table S1 Primers used for qPCR analysis**

| Gene name | Forward primer (5’→3’) | Reverse primer(5’→3’) |
| --- | --- | --- |
| *GA2* | CACATCAGATACTGGTCTTAGGC | GGAAGAATCCAAACAAATGAGT |
| *TIP41* | TGGAAGTTCAGAAGCAAGCCT | TCATCAGTATCCTCCCAACAAAA |

*GA2*: *ent*-kaurene synthase B; *TIP41*: tonoplast intrinsic protein.

**Table S2** **DEGs involved in the gibberellins biosynthetic pathway**

| ID | log2FC | description |
| --- | --- | --- |
| node_2193_length_6563_cov_65.007242_g1167_i0 | -0.836 | (loc_os04g52210.1 : 332.0) no description available & (at1g79460 : 185.0) Encodes for a protein with ent-kaurene synthase B activity which catalyzes the second step in the cyclization of GGPP to ent-kaurene in the gibberellins biosynthetic pathway. |
| node_47272_length_1984_cov_197.862899_g1167_i3 | -1.066 | (loc_os04g52210.1 : 332.0) no description available & (at1g79460 : 185.0) Encodes for a protein with ent-kaurene synthase B activity which catalyzes the second step in the cyclization of GGPP to ent-kaurene in the gibberellins biosynthetic pathway. |

## Supplementary Figures


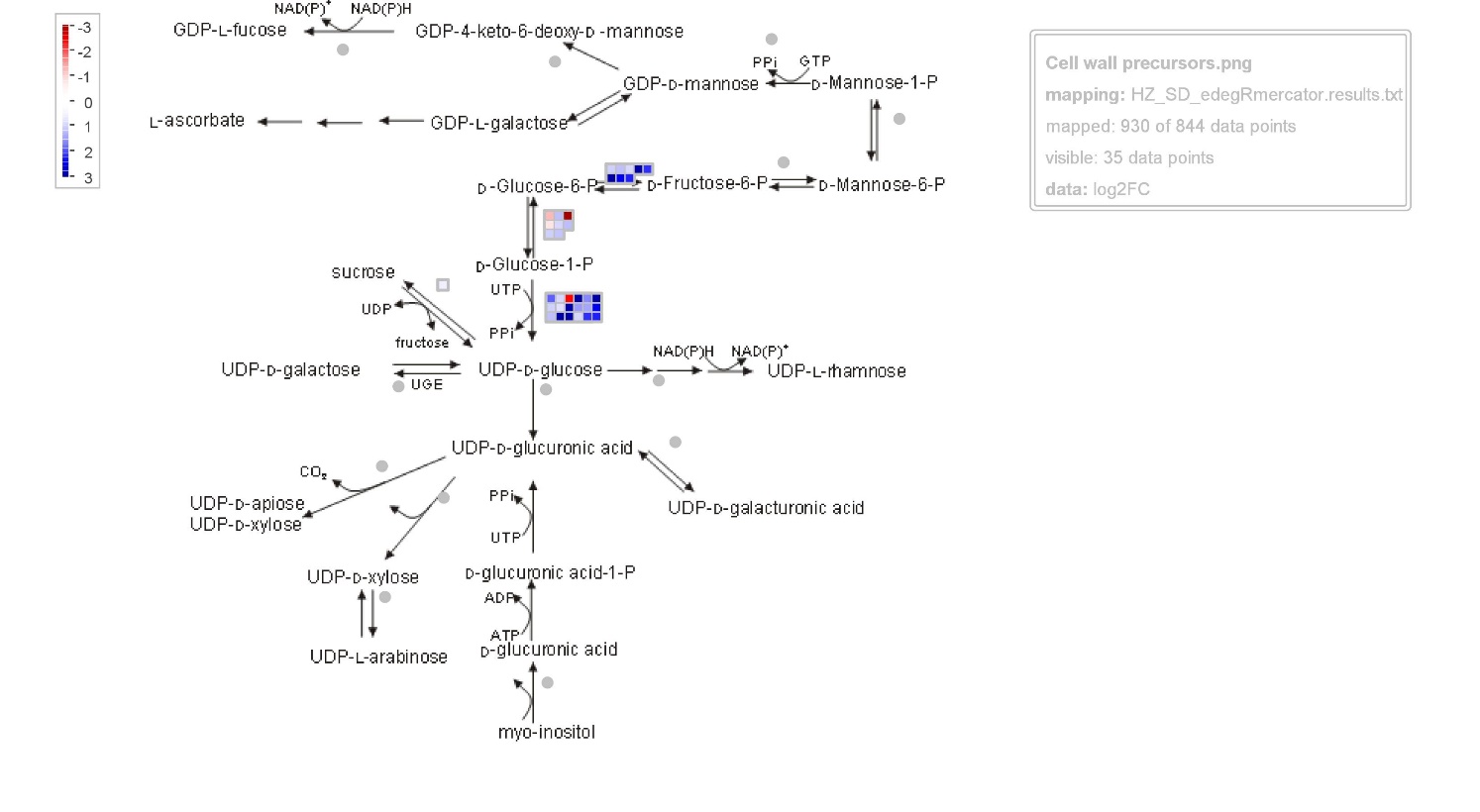


**Supplementary Figure 1.** DEGs involved in the cell wall precursors pathway. Each small square in the figure represents a gene.
